# Alteration of serum amino acid profiles by dietary adenine supplementation inhibits fatty liver development in rats

**DOI:** 10.1038/s41598-020-79234-w

**Published:** 2020-12-17

**Authors:** Hiroki Nishi, Daisuke Yamanaka, Masato Masuda, Yuki Goda, Koichi Ito, Fumihiko Hakuno, Shin-Ichiro Takahashi

**Affiliations:** 1 Department of Animal Sciences and Applied Biological Chemistry, Graduate School of Agriculture and Life Sciences, The University of Tokyo, 1-1-1 Yayoi, Bunkyo-ku, Tokyo, Japan; 2 Department of Veterinary Medical Sciences, Graduate School of Agriculture and Life Sciences, The University of Tokyo, Tokyo, Japan

**Keywords:** Endocrine system and metabolic diseases, Biomarkers, Predictive markers

## Abstract

Studies on animal models have demonstrated that feeding a low-arginine diet inhibits triacylglycerol (TAG) secretion from the liver, resulting in marked fatty liver development in rats. Here, we first showed that culturing hepatocytes in the medium mimicking the serum amino acid profile of low-arginine diet-fed rats induced TAG accumulation in the cells, indicating that the specific amino acid profile caused TAG accumulation in hepatocytes. Dietary adenine supplementation completely recovered hepatic TAG secretion and abolished hepatic TAG accumulation in rats. A comprehensive non-linear analysis revealed that inhibition of hepatic TAG accumulation by dietary adenine supplementation could be predicted using only serum amino acid concentration data. Comparison of serum amino acid concentrations indicated that histidine, methionine, and branched-chain amino acid (BCAA) concentrations were altered by adenine supplementation. Furthermore, when the serum amino acid profiles of low-arginine diet-fed rats were altered by modifying methionine or BCAA concentrations in their diets, their hepatic TAG accumulation was abolished. Altogether, these results suggest that an increase in methionine and BCAA levels in the serum in response to dietary arginine deficiency is a key causative factor for hepatic TAG accumulation, and dietary adenine supplementation could disrupt this phenomenon by altering serum amino acid profiles.

## Introduction

Nutritional status of macronutrients significantly affects systemic metabolism of animals, and their imbalanced intake leads to a series of metabolic diseases such as non-alcoholic fatty liver disease (NAFLD). NAFLD is defined as a series of hepatic diseases resulting from hepatic steatosis that develops without apparent excessive alcohol consumption. Among individuals with NAFLD, many remain under benign conditions (non-alcoholic fatty liver [NAFL]), whereas others exhibit non-alcoholic steatohepatitis (NASH) which can progress to liver inflammation, cirrhosis, and ultimately hepatocellular carcinoma^1,2^. Thus, an epidemic of NAFLD and NASH has increasingly given rise to clinical concerns worldwide.

For decades, the dietary imbalance of not only lipids or sugars but also other nutrients, including proteins/amino acids and nucleobases, has been reported to have significant impacts on neutral lipid accumulation in the liver, both in rodent models and in humans^2–5^. Because the detailed molecular mechanisms of NAFL progression have not yet been elucidated, there is no established definitive treatment for NAFLD; but dietary supplementation with some nutrients or small compounds is currently recommended^3^. For example, the purine nitrogenous base adenine can completely inhibit hepatic triacylglycerol (TAG) accumulation induced by a low-arginine diet, and the pyrimidine derivative orotic acid promotes fatty liver in rats^6,7^. Since it is known that dietary arginine deficiency alters nucleotide metabolism in the liver, thereby increasing orotic acid biosynthesis^8,9^, some researchers have raised the possibility that fatty liver induced by a low-arginine diet may be caused by increased orotic acid^10^. However, in this case too, the detailed molecular mechanisms still remain unclear as to how modified nucleotide metabolism or the related metabolites alter the hepatic lipid metabolism.

Many factors such as obesity, genetic background, viral infections, and dietary habits have been associated with NAFLD progression, but most of its underlying mechanisms and biomarkers for diagnosis have not been defined due to its high complexity^2,3,11,12^. A previous study demonstrated that a low-protein/amino acid diet and a low-arginine diet resulted in significant fatty liver development^4,5^, which led to the assumption that dietary arginine intake could have an important role in NAFLD progression. However, dietary arginine intake did not correlate with hepatic TAG levels, and a low-amino acid diet and a low-arginine diet induced fatty liver through a different mechanism, although both diets contained the same amounts of arginine^13^. This suggests that dietary amino acid concentrations are not primarily important; instead, serum amino acids are associated with hepatic lipid metabolism. In a previous study, a mathematical non-linear analysis based on a machine learning method revealed that a comprehensive serum amino acid profile, but not individual amino acid concentrations, correlated well with hepatic TAG levels in a rat model^5^.

Currently, many reports have suggested that serum amino acid profiles can be used as clinical diagnostic markers, since there is evidence for serum concentrations of some amino acids becoming up- or down-regulated in particular physiological disorders such as obesity, diabetes mellitus, some cancer types, and NAFLD^14–17^. We also demonstrated the correlation between serum amino acid profiles and hepatic TAG levels^5^. These results indicate that there is a strong link between serum amino acid profiles and hepatic lipid metabolism. However, since the available reports have used correlation analyses, we cannot determine whether the change in the serum amino acid profile is the cause or consequence of the disorder in question. Therefore, in the present study, we aimed to evaluate the possible role of serum amino acids in hepatic lipid accumulation by using our original methods: a cell-culture model and a non-linear machine leaning analysis. Furthermore, we illustrated how nucleobases are associated with serum amino acid profiles and affect lipid accumulation in the liver.

## Results

### A specific serum amino acid profile causes TAG accumulation in hepatocytes in a cell-autonomous manner

**Figure 1:**
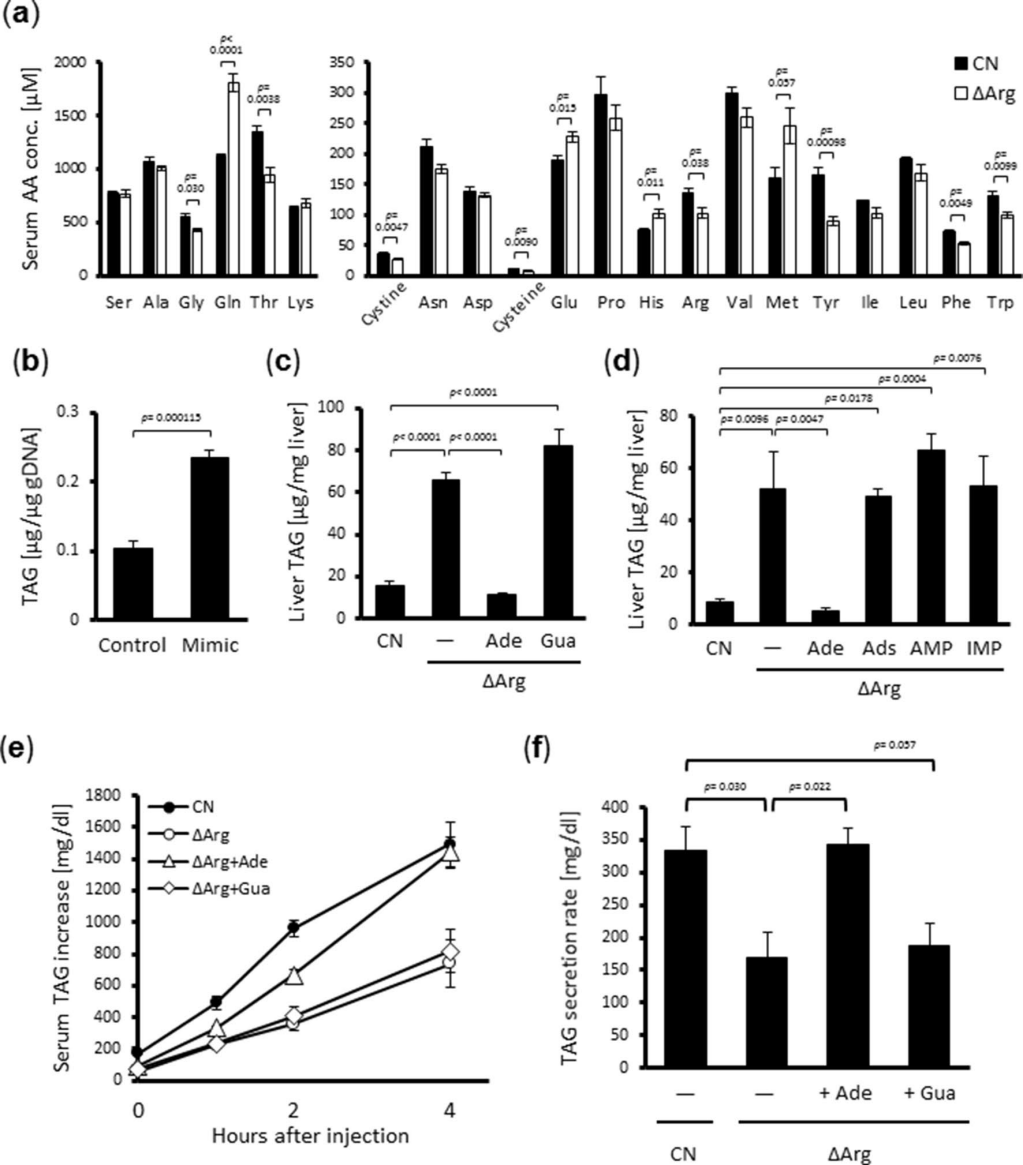
Dietary adenine supplementation reverses arginine deficient diet-induced lipid accumulation in the liver. (**a**) Six-week-old male Wistar rats were fed a control amino acid-mixed diet (CN) or low-arginine diet (ΔArg) for seven days, then bloods from the carotid artery were collected following 1 h fast. Sera were prepared and their amino acid concentrations were measured by LC–MS/MS. (**b**) H4IIE rat hepatoma cells were cultured for 24 h in a control medium or a medium mimicking serum amino acid composition of ΔArg diet-fed rats. TAG levels in the cell were measured. (**c**, **d**) Six-week-old male Wistar rats were fed the CN diet or ΔArg diet with or without adenine (Ade), guanine (Gua) (**c**), adenosine (Ads), adenosine-monophosphate (AMP) or inosine-monophosphate (IMP) (**d**) supplementation at the concentration of 3 g/kg for seven days. TAG levels in their livers were measured. (**e**, **f**) Six-week-old male Wistar rats were fed a CN diet or ΔArg diet with or without 3 g/kg adenine or guanine supplementation for seven days. After 16 h fasting on the last day, tyloxapol was administered intravenously, and serum TAG level was measured sequentially 0 (before injection), 1, 2, and 4 h after the reagent injection (**e**). The increasing rate of serum TAG level, which represents a TAG secretion rate from the liver, was calculated based on the regression line (**f**). [means ± S.E.M., (**a**, **c**, **d**: n = 5; **b**: n = 4; **e**, **f**: n = 6; Student’s t-test for **a**, **b**; Tukey–Kramer test for **c**, **d**, **f**)].

As previously reported^5^, a low-arginine (ΔArg) diet significantly induced TAG accumulation in the livers of rats in seven days, and serum amino acid profiles correlated well with liver TAG levels. Therefore, to evaluate the roles of serum amino acids in liver TAG accumulation, we compared the serum amino acid concentrations between ΔArg diet-fed rats and those fed a control diet. According to our results, the ΔArg diet increased or tended to increase the concentrations of four amino acids (methionine, histidine, glutamine, and glutamic acid), and reduced the concentrations of seven amino acids (cysteine/cystine, tryptophan, phenylalanine, tyrosine, arginine, threonine, and glycine), when compared to a control diet (Fig. 1a).

Next, we examined whether this specific serum amino acid profile in response to a ΔArg diet triggered TAG accumulation in the liver. For this, a culture medium that mimicked the serum composition of ΔArg diet-fed rats was prepared (see Supplementary Table S1). To this ΔArg-mimic medium, twice as much of the four up-regulated amino acids mentioned above as the control medium was added, and the seven down-regulated amino acids were not added. H4IIE rat hepatoma cells were cultured in control or ΔArg-mimic medium for 24 h, and the total TAG content was measured. The results demonstrated that the ΔArg-mimic medium significantly enhanced TAG accumulation in the cells when compared to the control medium (Fig. 1b), indicating that the specific amino acid profile of the hepatocytes’ surrounding environment could directly cause TAG accumulation. This also indicated that TAG accumulated in a cell-autonomous manner and under serum-free conditions.

### Adenine supplementation restores TAG secretion in the liver of ΔArg diet-fed rats

It has been reported that adenine supplementation in a ΔArg diet completely abolishes TAG accumulation in the liver (Fig. 1c, Supplementary Table S2)^6^. Therefore, we tested the effects of adenine and purine metabolites on hepatic TAG accumulation in a rat model. Interestingly, among purine nitrogenous bases (adenine, guanine) and adenine-related metabolites (adenosine, adenosine monophosphate [AMP], and inosine monophosphate [IMP]), only adenine supplementation inhibited hepatic TAG accumulation (Fig. 1c,d, Supplementary Table S3). While a ΔArg diet inhibited hepatic TAG secretion, resulting in fatty liver^13^, our results indicated that dietary adenine, but not guanine, fully rescued TAG secretion in the liver (Fig. 1e, f).

### Hepatic purine metabolism cannot explain the mechanism of fatty liver prevention by dietary adenine supplementation

**Figure 2:**
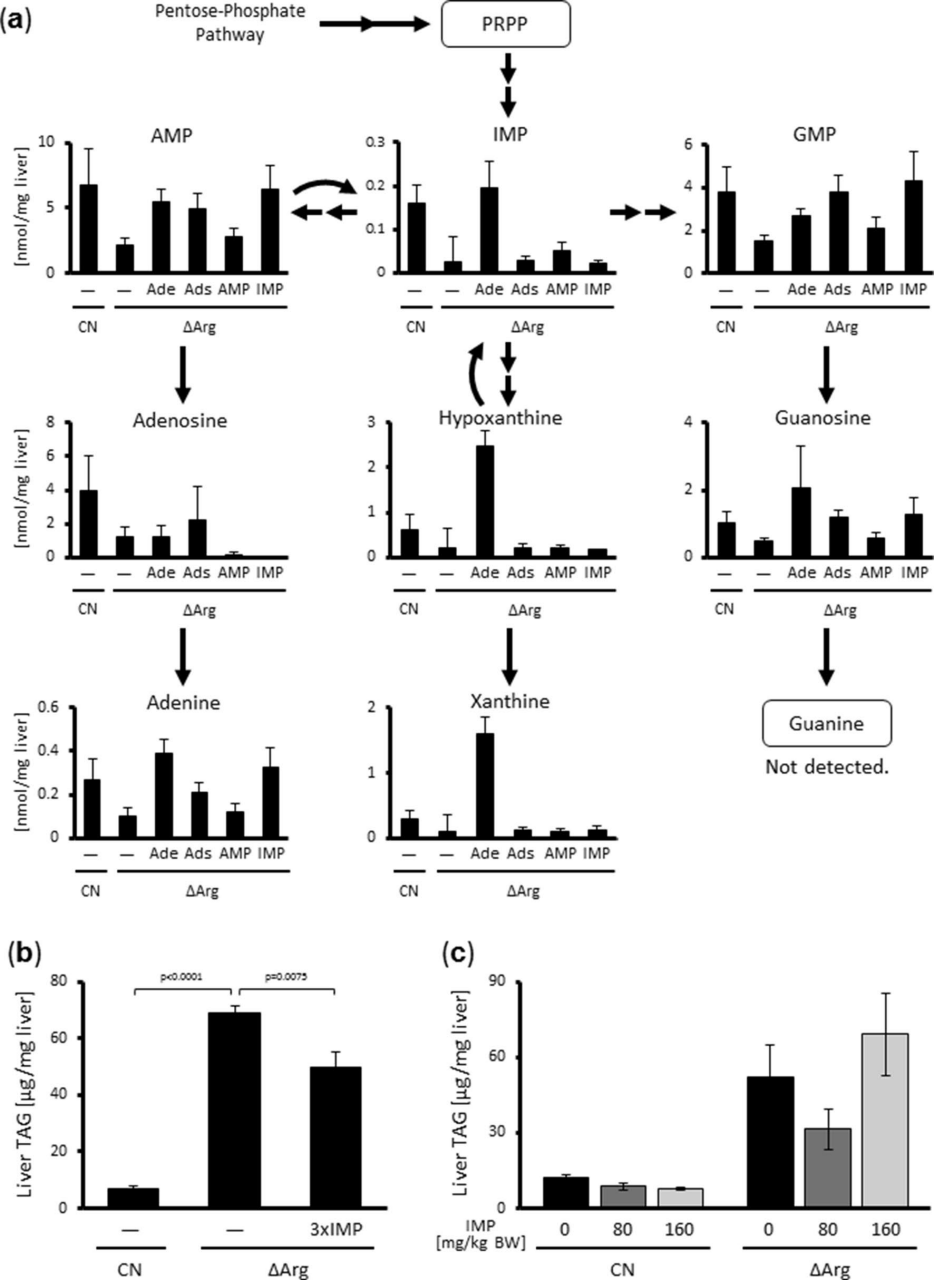
Adenine and related metabolites per se are unlikely to be involved in the hepatic lipid metabolic regulation. (**a**) Schematic diagram of purine metabolism and the levels of each metabolite in the livers of rats shown in Fig. 1d. (**b**) Six-week-old male Wistar rats were fed the CN diet or ΔArg diet with or without 9 g/kg IMP supplement for seven days. TAG levels in their livers were measured. (**c**) Six-week-old male Wistar rats were fed the CN diet or ΔArg diet for seven days. 80 or 160 mg/kg body wt. of IMP solution or just saline was injected via the tail vein at day 1, 3, and 5 after beginning of experimental diets. TAG levels in the livers were measured.

To evaluate the effects of dietary arginine deficiency and the supplementation of adenine and related molecules on hepatic purine metabolism, liver samples from rats in Fig. 1d were used in the analysis of related metabolites (Fig. 2a). Interestingly, supplementation with a certain compound did not necessarily increase the concentration of its corresponding metabolite in the liver, and hepatic adenine levels did not correlate with hepatic TAG levels, suggesting that hepatic adenine per se was not very significant for fatty liver progression. In contrast, dietary adenine supplementation increased hepatic IMP levels, which negatively correlated with liver TAG levels, while no other tested metabolite displayed any correlation (Fig. 2a).

In light of these results, we evaluated rats fed a diet containing three times as much IMP or those received IMP injection directly into the tail vein. The strong dietary IMP supplementation only slightly decreased ΔArg diet-induced hepatic TAG accumulation, while the direct intravenous injection did not show any significant effects on hepatic TAG levels (Fig. 2b,c). Collectively, these results indicate that the inhibition of ΔArg diet-induced fatty liver caused by dietary adenine occurs through a mechanism that cannot be explained by changes in purine metabolite levels in the liver.

### Multi-layer perceptron (MLP) analysis can predict the suppression of TAG accumulation only using serum amino acid profiles

In an earlier study from our group, we evaluated 135 data sets with serum amino acid concentrations and hepatic TAG levels in rats fed 27 different diets with varying amino acid compositions. A machine learning program based on MLP was established to predict individual hepatic TAG levels using the data on serum amino acid concentrations^5^. Based on this method, serum amino acid profiles and hepatic TAG levels were shown to exhibit a good correlation. Therefore, we analyzed the serum amino acid profiles of rats fed the purine-supplemented diets in order to predict their hepatic TAG accumulation levels by using this method.

**Figure 3:**
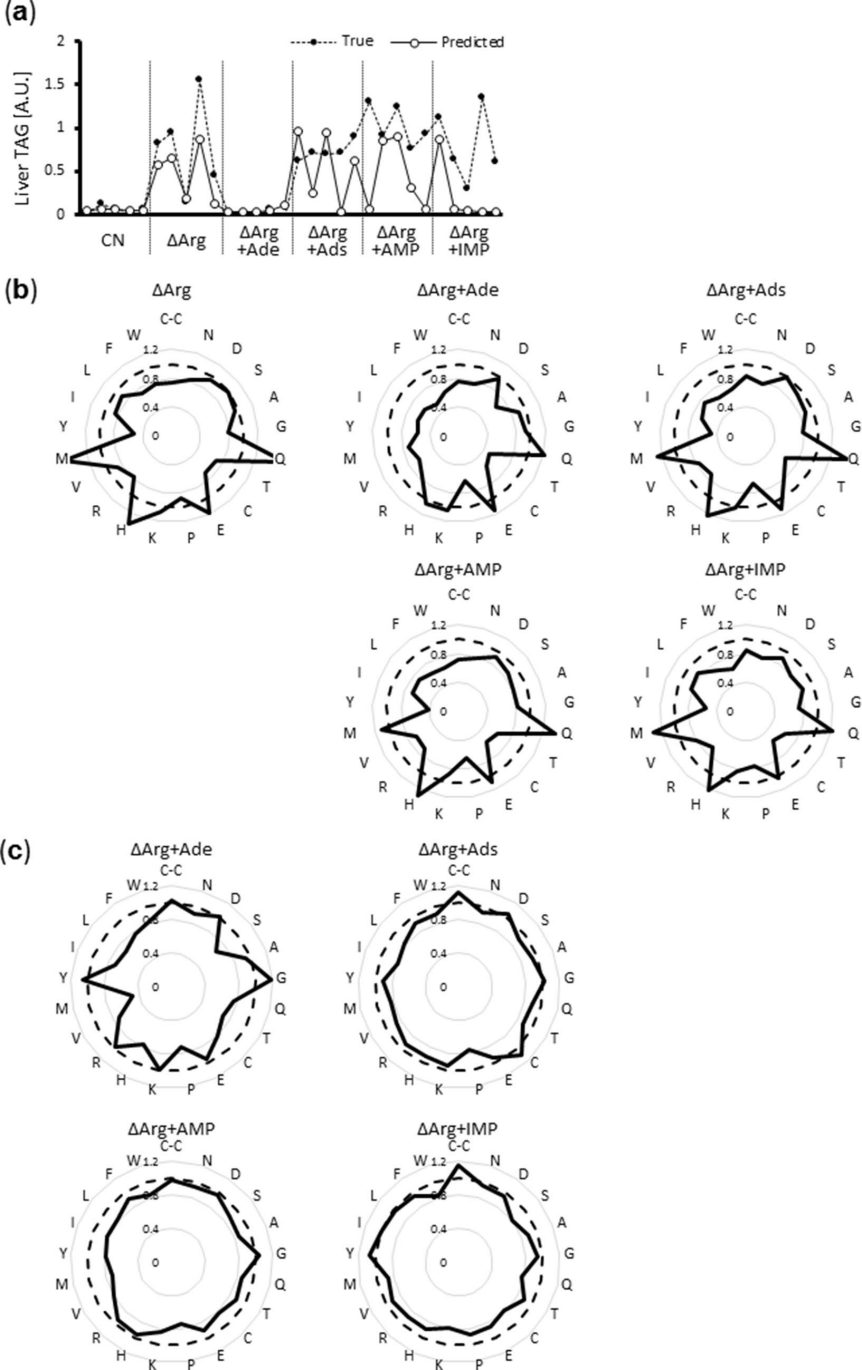
Dietary adenine affects hepatic lipid metabolism through modification of serum amino acid profile. (**a**) TAG levels in the individual liver of rats shown in Fig. 1d was estimated based on the data of their serum amino acid concentrations by multilayer perceptron (MLP). See Method section and reference^5^ for detail. Briefly, learning was conducted using serum amino acid concentrations of 95 rats as input data and putting corresponding measured liver TAG level in the output. All values used in this calculation were standardized and presented in the arbitrary unit. (**b**) Sera of rats shown in Fig. 1d were collected and their amino acid concentration was measured. Data are shown as a fold of the mean value of the CN group (dotted line), and average values of each experimental diet group (continuous line) are indicated. (**c**) The same data as (**b)**, but shown as a fold of the mean value of the ΔArg group (dotted line).

Figure 3a illustrates the program output of TAG values (“Predicted”) based only on the serum amino acid data and comparison with the measured hepatic TAG levels (“True”). Remarkably, the inhibitory effect of adenine supplementation on TAG accumulation was estimated with high accuracy, suggesting that adenine supplementation to the ΔArg diet would probably change the serum amino acid profile. Thus, we compared the serum amino acid profiles of each rat (ΔArg, ΔArg + adenine, ΔArg + adenosine, ΔArg + AMP, and ΔArg + IMP), and found that the serum amino acid profiles of the “fatty liver group” (ΔArg, ΔArg + adenosine, ΔArg + AMP, ΔArg + IMP) were quite similar to each other, but that of the ΔArg + adenine group greatly differed (Fig. 3b, Supplementary Table S4). These data suggested that adenine supplementation to a ΔArg diet, but not that of the other metabolites, changed the serum amino acid profile towards a “non-inducible pattern” for hepatic lipid accumulation.

### Alterations in the serum amino acid profile prevent hepatic TAG accumulation caused by a ΔArg diet

**Figure 4:**
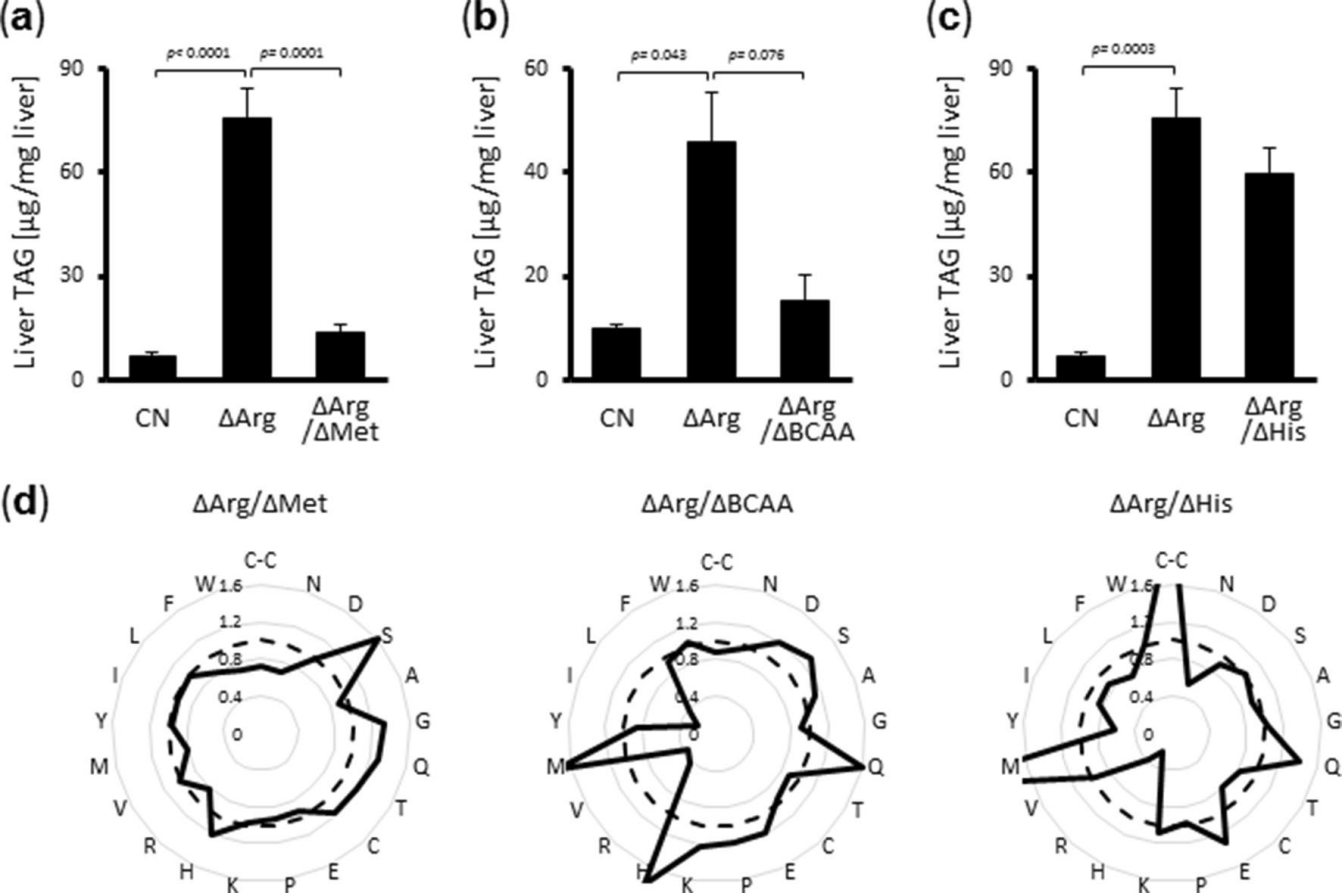
Perturbation of serum amino acid profile reverses arginine deficient diet-induced lipid accumulation in the liver. (**a–c**) Six-week-old male Wistar rats were fed the CN diet, ΔArg diet, both arginine and methionine deficient diet (ΔArg/ΔMet) (**a**), both arginine and BCAAs deficient diet (ΔArg/ΔBCAA) (**b**), or both arginine and histidine deficient diet (ΔArg/ΔHis) (**c**) for seven days. TAG levels in their livers. (**d**) Serum amino acid concentrations of rats shown in (**a–c**) were measured. Data of serum amino acid concentration are shown as a fold of the mean value of the CN group (dotted line), and average values of each experimental diet group (continuous line) are indicated. [bar; means ± S.E.M., (n = 4), Tukey–Kramer test].

Finally, to confirm the causative effect of serum amino acid profiles on hepatic TAG accumulation in vivo, we conducted different dietary interventions with the aim of perturbating serum amino acid compositions. Comparing the serum amino acid concentrations of rats fed a ΔArg diet and a ΔArg + adenine diet, the levels of methionine, histidine, and branched-chain amino acids ([BCAAs]; valine, leucine, isoleucine) were found to be much lower in ΔArg + adenine-fed rats (Fig. 3b,c). Subsequently, the concentration of each of these amino acids was reduced by two thirds in the ΔArg diet and rats were fed with this new diet composition for seven days. As expected, the deficiency of methionine or BCAAs in the ΔArg diet dramatically changed serum amino acid profiles and significantly abolished hepatic TAG accumulation, whereas histidine deficiency had little impact (Fig. 4, Supplementary Table S3, 4). Taken together, our results suggested that the specific serum amino acid profile formed in response to a ΔArg diet was able to regulate the lipid metabolism in hepatocytes, where attenuated TAG secretion caused TAG accumulation, leading to the development of fatty liver.

## Discussion

Dietary imbalance of some nutrients, including proteins/amino acids and nitrogenous bases, has been reported to have a significant impact on neutral lipid accumulation in the liver^3,6^. The amino acids, namely arginine, methionine, threonine, tryptophan, and glutamine has been suggested in NAFL^3,5,18^, but the underlying molecular mechanisms for this phenomenon have been described in a highly controversial manner with contradictory results. For example, while a methionine/choline-deficient diet is well known to induce NAFLD, a methionine-deficient diet prevents fatty liver progression in *ob/ob* mice^19,20^. Furthermore, a low-arginine or low-threonine diet induced fatty liver development in rats, whereas arginine or threonine supplementation to an amino acid-deficient diet failed to reverse fatty liver development^5^. These results strongly suggest that monitoring the intake of a single amino acid is not sufficient to understand the whole scenario of NAFL progression associated with amino acid nutrition. In this study, we provide another perspective for this relationship: the serum amino acid profile.

Adenine reportedly has an inhibitory effect on NAFL progression induced by a low-arginine diet, although the underlying mechanism is still unknown^6^. Some metabolites in purine metabolism, such as uric acid, are known as antioxidants; thus, it is possible that antioxidative properties of these molecules may have hepatoprotective effects^21^. If adenine itself is a bioactive molecule for controlling hepatic lipid metabolism, the ingested adenine should reach the liver through blood circulation. In addition, other purine metabolites should also change hepatic TAG levels because they could affect the adenine concentration through nucleotide metabolism. However, in the present study, even when adenine was supplemented in the diet, serum adenine concentrations were very low, often below the detectable level. Moreover, the hepatic adenine content did not correlate with hepatic TAG levels, although IMP actually increased adenine concentration in the liver (Fig. 2a). Since we previously discovered that dietary amino acid composition can dramatically affect serum amino acid profiles, changing it into an unexpected pattern^5^, we hypothesized that dietary adenine intake would also modify the serum amino acid profiles, which in turn would lead to a down-regulation of hepatic TAG accumulation. Indeed, oral adenine administration, but not other metabolites, modified serum amino acid profiles, and a medium amino acid composition that was similar to the serum of ΔArg diet-fed rats enhanced TAG accumulation in a cultured hepatocyte model (Fig. 1a,b). The media used in this study were serum-free, indicating that the amino acid profile was a sufficient signal for cellular metabolic regulation, regardless of any hormonal stimulation or neuronal input. These results illustrate a novel type of “metabolic regulatory amino acid signal” driven by comprehensive serum amino acid profiles.

In the current study, we used data of 20 protein-composing amino acids plus cystine for machine learning analyses, but it was not clear whether all these amino acids were indeed necessary for signal transduction. Although the ΔArg diet increased both methionine and histidine levels in the serum, a ΔArg/ΔHis diet did not exhibit an apparent effect on hepatic TAG levels, whereas a ΔArg/ΔMet diet significantly lowered these levels (Figs. 3b,c, 4). These results suggest that not all amino acids are essential to determine hepatic TAG levels. In addition, serum BCAA levels were not increased by a ΔArg diet, but were decreased by adenine supplementation, and hepatic TAG accumulation was inhibited by a ΔArg/ΔBCAA diet (Fig. 2b, 3b), implying that not only the absolute amino acid concentrations but also their relative concentrations are important. Furthermore, it was shown that the activities of peroxisome proliferator-activated receptor α (PPARα) and sterol regulatory element-binding protein-1c (SREBP1c), which are generally considered to be important transcription factors for the regulation of hepatic lipid metabolism, were not affected by amino acid deficiency^5^, suggesting that another mechanism is likely to be involved. Further research is necessary to understand which specific combinations of amino acids are actually functional and how these signals are recognized by the hepatocytes, leading to modifications in the activities of lipid metabolic machinery.

Altogether, we conclude that changes in comprehensive serum amino acid profiles in response to dietary amino acid compositions play a causative role in the regulation of lipid metabolism in the liver, and dietary adenine interferes with this signal transmission by perturbating serum amino acid profiles. This discovery should provide a novel insight into NAFLD pathophysiology. Although there are several exceptions , such as hepatitis C virus-induced NAFLD, which is unlikely to have a direct link with serum amino acids^22^, a certain part of the mechanism of pathogenesis should be explained by the amino acid signals that we discovered. This will lead to the design of useful diagnostic methods and establishment of effective prevention or treatment for NAFLD.

## Materials and methods

### Materials

For animal experimental diets, the vitamin mixture, mineral mixture, cellulose powder, and corn starch were purchased from Oriental Yeast Co. (Tokyo, Japan), while soybean oil was acquired from Nacalai Tesque (Tokyo, Japan). Solutions such as 10 × Earle’s buffered salt solution (EBSS) and 100 × MEM vitamin solution were purchased from Sigma-Aldrich (St. Louis, MO, USA). Dulbecco’s modified Eagle’s medium (DMEM) was purchased from Nissui Pharmaceutical Co. (Tokyo, Japan). Fetal bovine serum (FBS) was obtained from Sigma-Aldrich. Penicillin and streptomycin were obtained from Banyu Pharmaceutical Co., (Ibaraki, Japan). Other chemicals were of commercially available reagent grade.

### Animal experiments

Five-week-old male Wistar rats were purchased from Charles River Japan (Kanagawa, Japan). The rats were caged individually and maintained at 24 ± 1 °C with 50–60% humidity and a 12 h light/dark cycle (8:00–20:00/ 20:00–8:00). They were allowed free access to food and water during the experiment.

Prior to the experiments, all rats were fed normal chow for three days and a control (CN) diet containing 15% (w/w) amino acids (Supplementary Table S1) for the next four days as a prefeeding period. The animals were then divided into experimental groups. Thereafter, each group was given either the CN diet or each experimental diet for seven days. In a specific amino acid-deficient diet, the indicated amino acid concentration was 1/3 of that of the CN diet, and the concentration of the nucleotide supplement was 3 g/kg unless otherwise stated. For IMP injections, an inosine-5′-monophosphate disodium salt solution (100 mM, Sigma-Aldrich) was injected in the tail vein at days 1, 3, and 5 after beginning the experimental diets. In the control group, the same volume of saline solution was injected in the tail vein. All the diets were self-made. Throughout the experimental period, the body weight and food intake of all rats were measured at 10:00 AM every day. For tissue collection, rats were anesthetized with isoflurane (DS Pharma Animal Health, Tokyo, Japan) and then samples were obtained from the rats’ carotid arteries and livers. The serum was prepared soon after blood collection and liver samples were immediately frozen in liquid nitrogen. All samples were stored at -80 °C for future use.

All animal care and experiments conformed to the Guidelines for Animal Experiments of The University of Tokyo and were approved by the Animal Research Committee of The University of Tokyo.

### Cell culture and experiments

H4IIE-C3 cells (a rat hepatoma cell line, ATCC CRL-1600) were grown in DMEM supplemented with 10% FBS and antibiotics under 5% CO_2_ at 37 °C. Upon reaching sub-confluency, the medium was changed to an experimental serum-free medium (Supplementary Table S2), and the cells were further cultured for 24 h.

### Liver/cellular TAG measurement

Total lipids in the liver were extracted according to Folch’s method^23^ with small modifications. The frozen liver was homogenized in methanol:chloroform solution (1:2, v/v), followed by the addition of 20% volume of 0.8% KCl solution and centrifugation at 13,000 × *g*, at 4 °C for 10 min. Then, the organic (chloroform) layer was collected, the solvent was evaporated, and the remaining lipids were reconstituted in isopropanol. Similarly, lipids in cultured cells were extracted with a methanol:chloroform solution (1:1, v/v) and a saturated NaCl solution. The TAG content in the lipid extract was measured using Triglyceride E-test Wako (Wako, Osaka, Japan).

### MLP analysis

We built an MLP program using the libraries Keras, version 2.3.1, and TensorFlow, version 2.1.0, previously^5^, and we used the program in the present study with no modifications. The training data set was composed of serum amino acid profiles and liver TAG levels of each rat that were obtained in the same previous study. The MLP network configuration was as follows: a 21-dimensional input (20 amino acids + cystine), 4 hidden layers with 300, 300, 300, and 100 neurons from the input direction, and a 1-dimensional output of liver TAG levels. Dropouts were provided in each layer and set to 0.5, 0.4, 0.4, 0.4, and 0.3 from the input direction. The maximum learning epoch was set to 10,000. If the loss values did not update after more than 1,000 training epochs, “early stopping” was used to stop the training. Mean square error was used for the Loss function, and RMSprop for the optimization function. The activation function used ReLU in each layer. The trained model was used to predict liver TAG levels from untrained data sets of serum amino acid concentrations.

### Serum and liver metabolite analysis

For serum metabolite extraction, 50 µL of serum samples were mixed on ice with 120 μL methanol containing internal control substances: 25 μM 2-morpholinoethanesulfonic acid and 100 μM methionine sulfone. After centrifugation (16,000 × *g* for 10 min at 4 °C), 130 μL of supernatant was mixed with 250 μL ultrapure water and subjected to ultrafiltration using 3 kDa cutoff filters (Amicon Ultra 3 K device, Merck, Darmstadt, Germany), followed by 30 min of evaporation and 6 h of lyophilization.

For liver metabolite extraction, approximately 100 mg of liver pieces were homogenized in 500 μL of methanol containing internal controls. The homogenates were diluted with 250 μL of ultrapure water, and 600 μL of each homogenate was mixed with 400 μL chloroform. Following centrifugation (16,000 × *g* for 5 min at 4 °C), 800 μL of the upper phase was collected and subjected to 30 min of evaporation. Then, 300 μL of ultrapure water was added, followed by ultrafiltration and lyophilization.

The lyophilized metabolite samples were reconstituted in 200 μL ultrapure water, further diluted if necessary, and then subjected to LC–MS/MS (LCMS-8030, Shimadzu, Kyoto, Japan) analysis, using Method Package for Primary Metabolites ver. 2 (Shimadzu) according to the manufacturer’s protocol.

### TAG secretion assay

Six-week-old male Wistar rats were fed CN or ΔArg diets with or without nitrogenous base supplementation for seven days. Then, 200 mg/kg body weight of tyloxapol dissolved in 0.9% NaCl (Sigma-Aldrich) was administered into the tail vein (200 mg/kg body weight) under isoflurane anesthesia (DS Pharma Animal Health) and blood was collected 0, 1, 2, and 4 h after injection. TAG concentrations in the sera were measured using Triglyceride E-test Wako (Wako). Because tyloxapol is an inhibitor of endogenous lipoprotein lipase, TAG accumulation in the blood after its injection under fasting conditions reflects TAG secretion from the liver. Thus, the TAG increasing rate in the serum after tyloxapol injection was interpreted as the TAG secretion activity in the liver.

### Statistical analysis

Data are expressed as mean ± standard error of the mean (SEM), unless otherwise stated. Comparisons between two groups were performed using Student’s *t*-test. Comparisons among more than two groups were performed using one-way analysis of variance (ANOVA). If the *p* value obtained from the ANOVA test was < 0.05, the Tukey–Kramer post-hoc test was performed. Values of *p* < 0.05 were considered statistically significant. All statistical calculations were performed using JMP Pro (SAS Institute Inc., Cary, NC, USA).

## Supplementary information

Supplementary Tables.

## Data Availability

All data shown in this manuscript are available from the corresponding author on reasonable request.
