# Supplementary material for: Alteration of serum amino acid profiles by dietary adenine supplementation inhibits fatty liver development in rats
Source: Sci Rep. 2020 Dec 17;10:22110. doi: 10.1038/s41598-020-79234-w (PMC7747621; doi:10.1038/s41598-020-79234-w)
Supplement: Supplementary file 1 — Supplementary Tables. [file 41598_2020_79234_MOESM1_ESM.pdf]

# Alteration of serum amino acid profiles by dietary adenine supplementation inhibits fatty liver development in rats

Hiroki Nishi[1#], Daisuke Yamanaka[2#], Masato Masuda[1#], Yuki Goda[1], Koichi Ito[2], Fumihiko Hakuno[1]*, Shin-Ichiro Takahashi[1]

[1] Department of Animal Sciences and Applied Biological Chemistry,

[2] Department of Veterinary Medical Sciences, Graduate School of Agriculture and Life Sciences, The University of Tokyo, Tokyo, Japan

#These authors contributed equally to this work.

* **Corresponding author:** Fumihiko Hakuno, Ph.D., Departments of Animal Sciences and Applied Biological Chemistry, Graduate School of Agriculture and Life Sciences, The University of Tokyo, 1-1-1 Yayoi, Bunkyo-ku, Tokyo, Japan

TEL: +81-3-5841-1310; FAX: +81-3-5841-1311;
E-mail: hakuno@g.ecc.u-tokyo.ac.jp

**Supplementary Table S1. Compositions of experimental media.**

| | Control | ΔArg-mimic |
|---|---|---|
| Glycine | 30.0 | 0 |
| L-Alanine | 35.6 | 35.6 |
| L-Serine | 42.0 | 42.0 |
| L-Threonine | 95.0 | 0 |
| L-Cystine | 48.0 | 0 |
| L-Methionine | 30.0 | 60.0 |
| L-Glutamine | 584.0 | 1168.0 |
| L-Asparagine・$H_2O$ | 60.0 | 60.0 |
| L-Glutamic acid | 58.8 | 117.6 |
| L-Aspartic acid | 53.2 | 53.2 |
| L-Valine | 94.0 | 94.0 |
| L-Leucine | 105.0 | 105.0 |
| L-Isoleucine | 105.0 | 105.0 |
| L-Phenylalanine | 66.0 | 0 |
| L-Tyrosine | 72.4 | 0 |
| L-Tryptophan | 16.0 | 0 |
| L-Lysine・HCl | 146.0 | 146.0 |
| L-Arginine・HCl | 84.0 | 0 |
| L-Histidine | 31.0 | 62.0 |
| L-Proline | 46.0 | 46.0 |
| EBSS | 10% | 10% |
| vitamin solution | 1% | 1% |
| $NaHCO_3$ | 2.2 | 2.2 |
| D-glucose | 4.5 | 4.5 |
| antibiotics | | |

[mg/L]

**Supplementary Table S2. Compositions of experimental diets.**

| | CN | ΔArg | ΔArg/ΔMet | ΔArg/ΔBCAA | ΔArg/ΔHis |
|---|---|---|---|---|---|
| L-Isoleucine | 7.1 | 7.1 | 7.1 | 2.4 | 7.1 |
| L-Leucine | 13.0 | 13.0 | 13.0 | 4.3 | 13.0 |
| L-Lysine・HCl | 14.1 | 14.1 | 14.1 | 14.1 | 14.1 |
| DL-Methionine | 6.4 | 6.4 | 2.1 | 6.4 | 6.4 |
| L-Cystine | 0.8 | 0.8 | 0.8 | 0.8 | 0.8 |
| L-Phenylalanine | 7.2 | 7.2 | 7.2 | 7.2 | 7.2 |
| L-Tyrosine | 7.8 | 7.8 | 7.8 | 7.8 | 7.8 |
| L-Threonine | 6.1 | 6.1 | 6.1 | 6.1 | 6.1 |
| L-Tryptophan | 1.7 | 1.7 | 1.7 | 1.7 | 1.7 |
| L-Valine | 9.2 | 9.2 | 9.2 | 3.1 | 9.2 |
| L-Histidine | 4.1 | 4.1 | 4.1 | 4.1 | 1.4 |
| L-Arginine | 5.2 | 1.7 | 1.7 | 1.7 | 1.7 |
| L-Alanine | 4.1 | 4.1 | 4.1 | 4.1 | 4.1 |
| L-Aspartic acid | 5.1 | 5.1 | 5.1 | 5.1 | 5.1 |
| L-Asparagine・$H_2O$ | 5.8 | 5.8 | 5.8 | 5.8 | 5.8 |
| L-Glutamic acid | 14.6 | 14.6 | 14.6 | 14.6 | 14.6 |
| Glycine | 2.6 | 2.6 | 2.6 | 2.6 | 2.6 |
| L-Proline | 15.0 | 15.0 | 15.0 | 15.0 | 15.0 |
| L-Serine | 8.1 | 8.1 | 8.1 | 8.1 | 8.1 |
| L-Glutamine | 14.6 | 14.6 | 14.6 | 14.6 | 14.6 |
| cellulose | 100 | 100 | 100 | 100 | 100 |
| vitamin mixture | 10 | 10 | 10 | 10 | 10 |
| mineral mixtue | 40 | 40 | 40 | 40 | 40 |
| soybean oil | 50 | 50 | 50 | 50 | 50 |
| corn starch | 647.5 | 651.0 | 655.3 | 670.5 | 653.7 |
| total | 1000 | 1000 | 1000 | 1000 | 1000 |

[g/kg]

**Supplementary Table S3. Body weight and liver weight of rats subjected to each experiment.**
Initial and final body weight, and whole liver weight per final body weight of rats related to Fig. 1d, Fig. 4a, b, and c are shown as means ± S.E.M. Statistically significant differences are between values with the same alphabetic characters ($p < 0.05$).

| | | **Initial body weight [g]** | **Final body weight [g]** | **Liver weight [g/100 g BW]** |
|---|---|---|---|---|
| Fig. 1d | CN | 200.0 ± 3.0 | 256.6 ± 3.3 [a] | 5.1 ± 0.1 [a, b, c, d] |
| | ΔArg | 201.6 ± 2.7 | 251.6 ± 4.3 [b] | 6.4 ± 0.2 [a, e] |
| | ΔArg+Ade | 202.0 ± 2.9 | 230.4 ± 3.7 [a, b] | 5.4 ± 0.2 [e] |
| | ΔArg+Ads | 202.4 ± 2.1 | 250.8 ± 3.4 | 6.2 ± 0.2 [b] |
| | ΔArg+AMP | 201.8 ± 2.1 | 250.8 ± 3.9 | 6.2 ± 0.1 [c] |
| | ΔArg+IMP | 201.6 ± 2.3 | 257.2 ± 2.8 | 6.1 ± 0.1 [d] |
| Fig. 4a, c | CN | 217.3 ± 1.4 | 260.3 ± 4.6 | 5.0 ± 0.2 [a, b] |
| | ΔArg | 219.0 ± 3.4 | 270.0 ± 3.7 | 6.3 ± 0.2 [a, c] |
| | ΔArg/ΔMet | 215.3 ± 3.4 | 254.8 ± 4.1 | 4.9 ± 0.2 [c] |
| | ΔArg/ΔHis | 221.8 ± 5.2 | 267.0 ± 7.1 | 6.3 ± 0.2 [b] |
| Fig. 4b | CN | 181.3 ± 4.3 | 233.3 ± 2.9 [a] | 5.1 ± 0.2 |
| | ΔArg | 174.0 ± 4.9 | 224.0 ± 7.3 [b] | 5.1 ± 0.1 |
| | ΔArg/ΔBCAA | 167.3 ± 2.0 | 199.7 ± 1.0 [a, b] | 5.5 ± 0.2 |

**Supplementary Table S4. Serum amino acid concentrations of rats related to Figure 2b and 3d.**

Relative concentrations of serum amino acids measured by LCMS are shown as fold of mean value of CN group. These values were also used for MLP analysis of Fig. 3a as input vectors.

| | | Cystine (C-C) | Asn (N) | Asp (D) | Ser (S) | Ala (A) | Gly (G) | Gln (Q) | Thr (T) | Cys (C) | Glu (E) | Pro (P) | Lys (K) | His (H) | Arg (R) | Val (V) | Met (M) | Tyr (Y) | Ile (I) | Leu (L) | Phe (F) | Trp (W) |
|---|---|---|---|---|---|---|---|---|---|---|---|---|---|---|---|---|---|---|---|---|---|---|
| CN | #1 | 0.848 | 0.818 | 1.067 | 0.934 | 1.067 | 0.866 | 0.952 | 1.133 | 1.221 | 1.069 | 1.186 | 0.953 | 1.150 | 0.996 | 1.119 | 1.171 | 0.955 | 1.008 | 0.986 | 0.970 | 0.958 |
| | #2 | 0.947 | 1.084 | 1.059 | 1.072 | 1.013 | 0.824 | 1.032 | 0.872 | 0.730 | 1.091 | 1.126 | 1.052 | 1.041 | 0.813 | 1.038 | 1.139 | 1.293 | 1.040 | 1.058 | 1.105 | 1.190 |
| | #3 | 0.938 | 0.950 | 0.760 | 0.980 | 1.111 | 0.988 | 0.977 | 0.930 | 0.934 | 0.890 | 1.147 | 1.017 | 0.920 | 0.913 | 1.027 | 1.208 | 0.968 | 0.963 | 1.021 | 0.806 | 0.945 |
| | #4 | 1.116 | 1.207 | 1.081 | 0.958 | 0.923 | 1.091 | 0.986 | 1.116 | 0.960 | 0.924 | 0.973 | 1.037 | 0.901 | 1.197 | 0.941 | 0.934 | 0.840 | 1.015 | 0.943 | 0.974 | 1.074 |
| | #5 | 1.150 | 0.942 | 1.033 | 1.055 | 0.886 | 1.231 | 1.053 | 0.950 | 1.155 | 1.027 | 0.568 | 0.942 | 0.989 | 1.081 | 0.875 | 0.548 | 0.943 | 0.974 | 0.993 | 1.145 | 0.832 |
| ΔArg | #1 | 0.748 | 0.833 | 0.945 | 1.077 | 0.924 | 0.836 | 1.797 | 0.880 | 0.739 | 1.255 | 0.975 | 1.016 | 1.299 | 0.534 | 0.855 | 2.134 | 0.490 | 0.613 | 0.738 | 0.631 | 0.852 |
| | #2 | 0.628 | 0.910 | 0.989 | 1.029 | 0.943 | 0.725 | 1.678 | 0.658 | 0.727 | 1.052 | 1.110 | 1.048 | 1.596 | 0.670 | 1.020 | 1.905 | 0.396 | 0.892 | 1.006 | 0.692 | 0.822 |
| | #3 | 0.720 | 0.676 | 0.864 | 0.832 | 0.903 | 0.670 | 1.363 | 0.637 | 0.685 | 1.237 | 0.666 | 0.806 | 1.082 | 0.865 | 0.702 | 1.140 | 0.501 | 0.732 | 0.723 | 0.737 | 0.691 |
| | #4 | 0.762 | 0.828 | 0.921 | 1.030 | 0.923 | 0.824 | 1.496 | 0.749 | 0.696 | 1.115 | 0.784 | 1.180 | 1.596 | 0.920 | 0.799 | 1.218 | 0.592 | 0.831 | 0.812 | 0.679 | 0.639 |
| | #5 | 0.844 | 0.864 | 1.036 | 0.983 | 1.048 | 0.855 | 1.738 | 0.582 | 0.617 | 1.323 | 0.820 | 1.250 | 1.240 | 0.813 | 0.973 | 1.251 | 0.695 | 1.117 | 1.099 | 0.871 | 0.772 |
| ΔArg +Ade | #1 | 0.703 | 0.627 | 0.979 | 0.636 | 0.954 | 0.859 | 1.251 | 0.723 | 0.610 | 1.074 | 0.896 | 1.244 | 1.159 | 0.635 | 0.780 | 1.043 | 0.566 | 0.794 | 0.630 | 0.400 | 0.780 |
| | #2 | 0.585 | 0.739 | 0.975 | 0.692 | 0.931 | 0.884 | 1.093 | 0.485 | 0.487 | 1.046 | 0.698 | 0.987 | 1.269 | 0.626 | 0.602 | 1.004 | 0.712 | 0.488 | 0.580 | 0.596 | 0.647 |
| | #3 | 0.851 | 0.866 | 1.083 | 0.683 | 0.918 | 0.832 | 1.350 | 0.410 | 0.451 | 1.417 | 0.650 | 1.316 | 1.093 | 0.703 | 0.506 | 0.709 | 0.575 | 0.414 | 0.381 | 0.369 | 0.560 |
| | #4 | 0.802 | 0.692 | 0.918 | 0.568 | 0.691 | 0.924 | 1.152 | 0.389 | 0.628 | 0.991 | 0.351 | 0.834 | 0.657 | 0.835 | 0.615 | 0.367 | 0.495 | 0.583 | 0.680 | 0.697 | 0.515 |
| | #5 | 0.863 | 0.829 | 0.894 | 0.727 | 1.015 | 1.115 | 1.189 | 0.455 | 0.623 | 1.193 | 0.549 | 0.865 | 1.008 | 0.938 | 0.653 | 0.508 | 0.480 | 0.760 | 0.736 | 0.655 | 0.659 |
| ΔArg +Ads | #1 | 0.723 | 0.717 | 0.878 | 0.837 | 0.893 | 0.761 | 1.476 | 0.538 | 0.759 | 0.957 | 0.622 | 0.783 | 1.146 | 0.583 | 0.717 | 1.324 | 0.330 | 0.532 | 0.612 | 0.618 | 0.605 |
| | #2 | 0.875 | 0.692 | 0.904 | 0.893 | 0.939 | 0.718 | 1.440 | 0.677 | 0.782 | 1.145 | 0.889 | 0.795 | 1.168 | 0.599 | 0.933 | 1.776 | 0.530 | 0.656 | 0.775 | 0.605 | 0.908 |
| | #3 | 0.889 | 0.859 | 1.118 | 0.997 | 0.953 | 0.864 | 1.501 | 0.657 | 0.940 | 1.153 | 0.826 | 1.142 | 1.425 | 0.782 | 0.776 | 1.489 | 0.547 | 0.714 | 0.735 | 0.593 | 0.641 |
| | #4 | 0.939 | 0.884 | 1.076 | 0.994 | 0.948 | 0.950 | 1.425 | 0.668 | 0.800 | 1.059 | 0.568 | 1.284 | 1.406 | 0.854 | 0.716 | 0.939 | 0.556 | 0.667 | 0.807 | 0.827 | 0.738 |
| | #5 | 0.709 | 0.651 | 0.982 | 0.816 | 0.703 | 0.668 | 1.396 | 0.555 | 0.538 | 1.267 | 0.405 | 0.987 | 1.047 | 0.742 | 0.622 | 0.790 | 0.456 | 0.685 | 0.722 | 0.650 | 0.517 |
| ΔArg +AMP | #1 | 0.914 | 0.727 | 0.986 | 0.846 | 0.637 | 0.789 | 1.279 | 0.633 | 0.547 | 1.136 | 0.487 | 0.979 | 1.239 | 0.802 | 0.507 | 0.809 | 0.387 | 0.545 | 0.530 | 0.617 | 0.679 |
| | #2 | 0.679 | 0.818 | 0.848 | 0.902 | 0.863 | 0.783 | 1.215 | 0.600 | 0.606 | 0.909 | 0.765 | 0.870 | 1.230 | 0.699 | 0.768 | 1.103 | 0.388 | 0.638 | 0.645 | 0.593 | 0.590 |
| | #3 | 0.584 | 0.794 | 0.941 | 0.849 | 0.803 | 0.662 | 1.397 | 0.710 | 0.591 | 1.024 | 0.815 | 0.963 | 1.509 | 0.533 | 0.755 | 1.756 | 0.572 | 0.699 | 0.877 | 0.782 | 0.673 |
| | #4 | 0.664 | 0.867 | 1.046 | 0.999 | 0.905 | 1.131 | 1.968 | 0.625 | 0.414 | 1.460 | 0.638 | 0.891 | 1.579 | 0.775 | 0.673 | 0.980 | 0.296 | 0.728 | 0.717 | 0.579 | 0.540 |
| | #5 | 0.786 | 0.649 | 0.783 | 0.749 | 0.842 | 0.697 | 1.006 | 0.565 | 0.713 | 0.872 | 0.563 | 0.719 | 0.961 | 0.738 | 0.726 | 0.838 | 0.441 | 0.809 | 0.791 | 0.709 | 0.684 |
| ΔArg +IMP | #1 | 0.793 | 0.776 | 0.848 | 0.818 | 0.831 | 0.567 | 1.291 | 0.586 | 0.406 | 1.062 | 0.761 | 0.795 | 1.470 | 0.502 | 0.916 | 1.951 | 0.546 | 0.891 | 0.929 | 0.693 | 0.728 |
| | #2 | 0.661 | 0.657 | 0.845 | 0.738 | 0.805 | 0.622 | 1.087 | 0.562 | 0.564 | 0.887 | 0.789 | 0.760 | 1.009 | 0.645 | 0.713 | 1.187 | 0.656 | 0.691 | 0.724 | 0.562 | 0.532 |
| | #3 | 0.748 | 0.611 | 0.834 | 0.766 | 0.811 | 0.804 | 1.106 | 0.739 | 0.588 | 1.044 | 0.890 | 0.782 | 0.933 | 0.669 | 0.823 | 1.178 | 0.648 | 0.826 | 0.840 | 0.700 | 0.611 |
| | #4 | 1.125 | 1.126 | 0.989 | 0.934 | 1.024 | 0.729 | 1.424 | 0.816 | 0.596 | 1.176 | 0.919 | 1.027 | 1.638 | 0.818 | 0.948 | 1.406 | 0.596 | 1.058 | 1.046 | 0.799 | 0.712 |
| | #5 | 0.965 | 0.735 | 0.965 | 0.732 | 0.723 | 0.930 | 1.209 | 0.415 | 0.578 | 0.946 | 0.438 | 0.791 | 1.028 | 0.761 | 0.592 | 0.859 | 0.391 | 0.674 | 0.742 | 0.695 | 0.530 |
| ΔArg /ΔMet | #1 | 0.921 | 0.451 | 1.140 | 1.670 | 0.899 | 1.295 | 1.438 | 1.368 | 1.441 | 0.977 | 1.043 | 1.099 | 1.416 | 0.579 | 1.111 | 1.504 | 1.206 | 0.986 | 1.107 | 0.940 | 0.938 |
| | #2 | 0.899 | 0.758 | 0.935 | 1.551 | 0.853 | 1.037 | 1.270 | 1.281 | 1.151 | 0.934 | 0.998 | 0.928 | 1.178 | 0.752 | 1.130 | 0.806 | 1.196 | 1.076 | 1.076 | 0.769 | 0.804 |
| | #3 | 0.521 | 0.791 | 0.928 | 1.699 | 0.805 | 1.770 | 1.261 | 1.207 | 1.235 | 0.959 | 0.791 | 1.017 | 1.321 | 1.046 | 0.864 | 0.597 | 0.615 | 0.912 | 0.901 | 0.765 | 0.548 |
| | #4 | 0.582 | 0.789 | 0.775 | 1.554 | 0.996 | 1.197 | 1.169 | 0.895 | 0.868 | 0.836 | 0.925 | 0.834 | 0.945 | 0.846 | 0.949 | 0.369 | 0.852 | 0.905 | 0.846 | 0.755 | 0.560 |
| ΔArg /ΔBCAA | #1 | 1.192 | 0.658 | 1.102 | 1.029 | 0.922 | 0.895 | 1.595 | 0.819 | 0.782 | 1.202 | 1.138 | 1.189 | 1.520 | 0.709 | 0.319 | 1.696 | 0.935 | 0.236 | 0.317 | 0.836 | 0.983 |
| | #2 | 0.939 | 1.027 | 1.350 | 1.498 | 1.444 | 0.791 | 1.780 | 1.046 | 0.743 | 1.277 | 1.472 | 1.152 | 2.027 | 0.144 | 0.344 | 2.868 | 0.869 | 0.170 | 0.362 | 1.045 | 1.007 |
| | #3 | 0.514 | 1.189 | 1.100 | 1.373 | 1.048 | 1.009 | 1.479 | 0.835 | 1.379 | 1.105 | 0.950 | 1.343 | 1.871 | 0.452 | 0.390 | 0.686 | 0.823 | 0.233 | 0.454 | 0.933 | 1.097 |
| ΔArg /ΔHis | #1 | 2.852 | 0.492 | 1.056 | 0.971 | 0.969 | 0.904 | 1.937 | 0.760 | 0.753 | 1.533 | 1.033 | 1.158 | 0.193 | 0.212 | 0.918 | 3.253 | 0.668 | 0.762 | 0.759 | 0.703 | 1.024 |
| | #2 | 2.615 | 0.238 | 0.568 | 1.119 | 0.930 | 0.898 | 1.064 | 1.053 | 0.827 | 1.444 | 1.020 | 0.884 | 0.344 | 0.457 | 1.039 | 3.715 | 0.532 | 0.896 | 0.898 | 0.708 | 1.068 |
| | #3 | 2.120 | 0.749 | 1.071 | 1.118 | 0.952 | 1.232 | 1.476 | 0.841 | 0.946 | 1.154 | 1.107 | 1.295 | 0.177 | 0.495 | 1.245 | 1.956 | 0.890 | 1.114 | 1.170 | 0.956 | 1.129 |
| | #4 | 1.824 | 0.701 | 0.882 | 0.810 | 0.799 | 1.072 | 1.116 | 0.654 | 0.638 | 1.084 | 0.706 | 0.951 | 0.182 | 0.346 | 0.633 | 1.274 | 0.431 | 0.590 | 0.622 | 0.626 | 0.759 |
